# Moonlight-driven biological choruses in Hawaiian coral reefs

**DOI:** 10.1371/journal.pone.0299916

**Published:** 2024-03-20

**Authors:** Daniel Duane, Simon Freeman, Lauren Freeman

**Affiliations:** 1 Naval Undersea Warfare Center, Newport, Rhode Island, United States of America; 2 Advanced Research Projects Agency–Energy, Washington, D.C., United States of America; University of Windsor, CANADA

## Abstract

Sounds from fish and invertebrates in coral reefs can create persistent cacophonies that can be recorded for ecosystem monitoring, including during nighttime hours where visual surveys are typically not feasible. Here we use soundscape measurements in Hawaii to demonstrate that multiple coral reef communities are rapidly responsive to shifts in nighttime ambient light, with sustained changes in biological sound between moonrise and moonset. High frequency pulse train sounds from fish (0.5-1.5 kHz) are found to increase during moonlight hours, while low frequency fish vocalizations (0.1-0.3 kHz) and invertebrate sounds (2-20 kHz) are found to decrease during moonlight hours. These discoveries suggest that the rising and setting of the moon triggers regular shifts in coral reef ecosystem interactions. Future acoustic monitoring of reef health may be improved by comparing soundscapes during moonlight and non-moonlight hours, which may provide early indicators of shifts in the relative abundance of separate reef communities.

## Introduction

Rising seawater temperatures and ocean acidification have led to the widespread degradation of coral reefs, with an estimated 50% decrease in global coral cover from 1950 to 2021 [1]. Effective conservation efforts require long-term monitoring of reef ecosystems, however conventional diver surveys may significantly undersample the environment and cannot be conducted at night when reef communities are most active [2, 3].

Acoustic monitoring is a reliable and noninvasive method of observing coral reef ecosystems, with increased noise from fish and invertebrates associated with healthier reefs [2, 4–6]. A single acoustic sensor in a reef may record hundreds of fish sounds and thousands of invertebrate sounds per minute, providing an aggregated measurement of biological activity which may allow for the early detection of changes in population abundance and behavior [7, 8]. Coral reef soundscapes also act as a beacon for recruiting pelagic fish and crustacean larva [9–11], and recent restoration efforts have been aided by artificial playback of healthy reef sounds to promote recruitment [12]. Given the potential for acoustic monitoring and playback to facilitate reef restoration efforts, it is important to understand how the interactions of separate reef communities contribute to changes in underwater soundscapes.

Here we use long-term acoustic measurements of coral reef soundscapes in Hawaii to demonstrate the rapid response of multiple reef communities to changes in nighttime ambient light after moonrise and moonset, in addition to previously-known biological responses to daylight [4, 13, 14]. High frequency pulse train sounds from fish (0.5–1.5 kHz) are found to increase during moonlight hours, while low frequency fish vocalizations (0.1–0.3 kHz) and invertebrate sounds (2–20 kHz) are found to decrease during moonlight hours. The observed changes in biological sound between moonrise and moonset are likely a response to changing lunar light levels rather than tidal variations, since high tide and low tide are not synchronized with the rising and setting of the moon in the environment studied (S1 Fig).

## Methods

**Fig 1:**
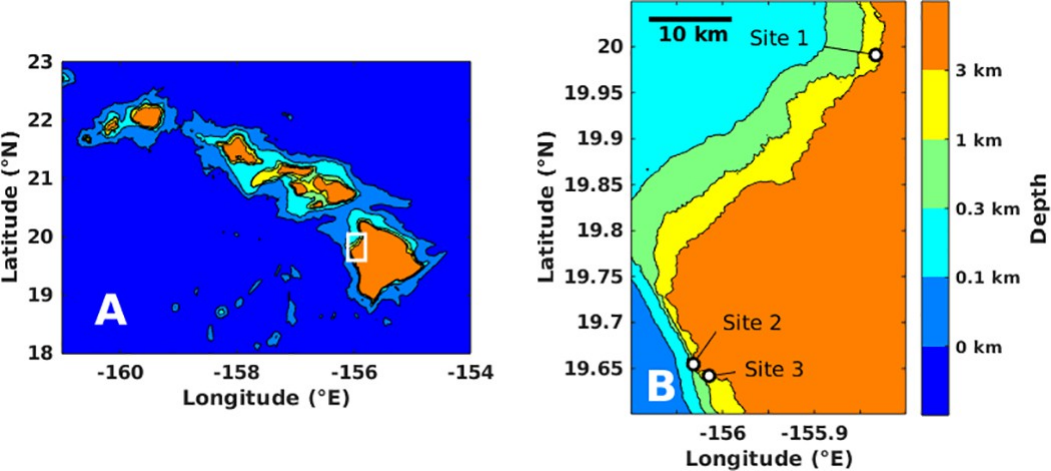
Survey locations. Bathymetric maps showing geographic coordinates of survey locations in Hawaii [15]. The white box in A denotes the region shown in B. Labeled white dots in B denote the location of the three survey sites.

Hydrophones were mounted on the seafloor in three coral reef environments off the west coast of Hawaii Island (Fig 1). Survey Site 1 is off the coast of Kawaihae Harbor, south of Hapuna Beach State Park, where the bottom is covered with spur and groove coral fingers alternating with sand channels. The sand is at about 30 m depth and the coral ridges rise up to about 15 m depth at the top. The sensor was placed in a sand channel at 21.9 m depth between two coral ridges. Survey Sites 2 and 3 are near a long reef slope ending in a sandy bottom at 20–35 m depth. The sensor at Site 2 is closer to the reef at 21.9 m depth, and the sensor at Site 3 is in deeper water along the sand slope at 33.2 m depth. Deployments lasted from 3/17/2020 to 5/15/2021 for Survey Site 1, 4/21/2020 to 9/6/2021 for Survey Site 2, and 4/21/2020 to 8/23/2021 for Survey Site 3. The sensor at Site 1 was replaced on 9/24/2020. A Loggerhead LS1 recorder was deployed at Site 1, and Loggerhead LS1X recorders were deployed at Sites 2 and 3.

Hydrophones were set to record at a sampling rate of 96 kHz, recording for 1 minute every 15 minutes in Survey Site 1, and 1 minute every 10 minutes in Survey Sites 2 and 3. These duty cycles are significantly shorter than the timescales of the environmental changes studied here. The sensitivity of the HTI96-min hydrophones used here was -170 dB re V/*μ*Pa, and the frequency range was 2 Hz to 30 kHz. Spectrograms of each one-minute recording were generated using fast-fourier transforms of 70% overlapping segments with 2048 samples windowed with a hann function.

Contributions to the soundscape from separate marine communities are isolated by selecting acoustic frequency bands typically dominated by specific biological sounds. Vocalizations from Hawaiian coral reef fish primarily occupy frequencies between 0.1–0.3 kHz [16], high frequency pulse train sounds occupy frequencies between 0.5–1.5 kHz [16], and broadband clicks from snapping shrimp and other invertebrates occupy frequencies between 2–20 kHz [17, 18].

The mean change in power spectral density due to the response of marine life to moonlight 〈ΔPSD〉 is measured across each month surveyed according to:

$$\begin{array}{c} \left\langle \Delta \text{PSD}\right\rangle =10\;{\text{log}}_{10}(\frac{1}{n_{\text{moon}}}\sum_{i=1}^{n_{\text{moon}}}S_{\text{moon},i})-10\;{\text{log}}_{10}(\frac{1}{n_{\text{no}\;\text{moon}}}\sum_{i=1}^{n_{\text{no}\;\text{moon}}}S_{\text{no}\;\text{moon},i}) \end{array}$$ (1)

where *S*_moon,*i*_ and *S*_no moon,*i*_ are the power spectral density in linear scale during nighttime moonlight and nighttime non-moonlight hours respectively, averaged in time across the *i*th one-minute sample and in frequency across the relevant band. Samples where the averaged power spectral density is in the top 2 percentile for each month are discarded in order to minimize the contribution of loud non-biological sounds such as ship-radiated noises. Moonlight hours are defined here as times between 1.5 hours after moonrise and 1.5 hours before moonset, and non-moonlight hours are defined as times between 1.5 hours after moonset and 1.5 hours before moonrise. Nighttime hours are defined here as times between 4 hours after sunset and 2 hours before sunrise, in order to avoid contributions from dusk and dawn choruses of fish and invertebrates. *n*_moon_ and *n*_no moon_ are respectively defined as the number of one-minute samples available during nighttime moonlight hours and nighttime non-moonlight hours in the month surveyed.

The standard deviation of the power spectral density during moonlight hours is calculated according to:

$$\begin{array}{c} \sigma_{\text{moon}}=\sqrt{\frac{1}{n_{\text{moon}}}\sum_{i=1}^{n_{\text{moon}}}({\text{PSD}}_{\text{moon},i}-{\left\langle \text{PSD}\right\rangle }_{\text{moon}}{)}^{2}} \end{array}$$ (2)

where

$$\begin{array}{c} {\text{PSD}}_{\text{moon},i}=10\;{\text{log}}_{10}\left(S_{\text{moon},i}\right) \end{array}$$ (3)

and

$$\begin{array}{c} {\left\langle \text{PSD}\right\rangle }_{\text{moon}}=10\;{\text{log}}_{10}(\frac{1}{n_{\text{moon}}}\sum_{i=1}^{n_{\text{moon}}}S_{\text{moon},i}) \end{array}$$ (4)

The standard deviation of the power spectral density during non-moonlight hours is similarly calculated according to:

$$\begin{array}{c} \sigma_{\text{no}\;\text{moon}}=\sqrt{\frac{1}{n_{\text{no}\;\text{moon}}}\sum_{i=1}^{n_{\text{no}\;\text{moon}}}({\text{PSD}}_{\text{no}\;\text{moon},i}-{\left\langle \text{PSD}\right\rangle }_{\text{no}\;\text{moon}}{)}^{2}} \end{array}$$ (5)

where

$$\begin{array}{c} {\text{PSD}}_{\text{no}\;\text{moon},i}=10\;{\text{log}}_{10}\left(S_{\text{no}\;\text{moon},i}\right) \end{array}$$ (6)

and

$$\begin{array}{c} {\left\langle \text{PSD}\right\rangle }_{\text{no}\;\text{moon}}=10\;{\text{log}}_{10}(\frac{1}{n_{\text{no}\;\text{moon}}}\sum_{i=1}^{n_{\text{no}\;\text{moon}}}S_{\text{no}\;\text{moon},i}) \end{array}$$ (7)

The standard deviation of the change in power spectral density due to the response of marine life to moonlight *σ*_ΔPSD_ is then defined as the pooled standard deviation of *σ*_moon_ and *σ*_no moon_:

$$\begin{array}{c} \sigma_{\Delta \text{PSD}}=\sqrt{\frac{\left(n_{\text{moon}}-1\right)\sigma_{\text{moon}}^{2}+\left(n_{\text{no}\;\text{moon}}-1\right)\sigma_{\text{no}\;\text{moon}}^{2}}{n_{\text{moon}}+n_{\text{no}\;\text{moon}}-2}} \end{array}$$ (8)

Statistical significance is evaluated for each full month surveyed and for each frequency band by comparing the mean change in power spectral density during moonlight hours 〈ΔPSD〉 to the standard deviation *σ*_ΔPSD_. If 〈ΔPSD〉 > *σ*_ΔPSD_, the change in power spectral density due to the response of marine life to moonlight is considered statistically significant for the month surveyed. If 〈ΔPSD〉 < *σ*_ΔPSD_, the difference is not considered statistically significant.

Measurements of 〈ΔPSD〉 and *σ*_ΔPSD_ are shown in S1 Table for the 0.5–1.5 kHz band associated with high frequency pulse trains from fish, S2 Table for the 0.1–0.3 kHz band associated with low-frequency fish vocalizations, and S3 Table for the 2–20 kHz band associated with invertebrate sounds. *n*_moon_ and *n*_no moon_ values are shown in S4 Table. Measurements of 〈ΔPSD〉 are not made in the 0.1–0.3 kHz and 0.5–1.5 kHz frequency bands between December and March, since humpback whale vocalizations dominate the underwater soundscape for the majority of these months. Measurements of 〈ΔPSD〉 are also not made in the 0.1–0.3 kHz and 0.5–1.5 kHz frequency bands after June 2021 in Site 2 or May 2021 in Site 3 since low-frequency (<2 kHz) electrical noise contaminated the signal during these months.

## Results

**Fig 2:**
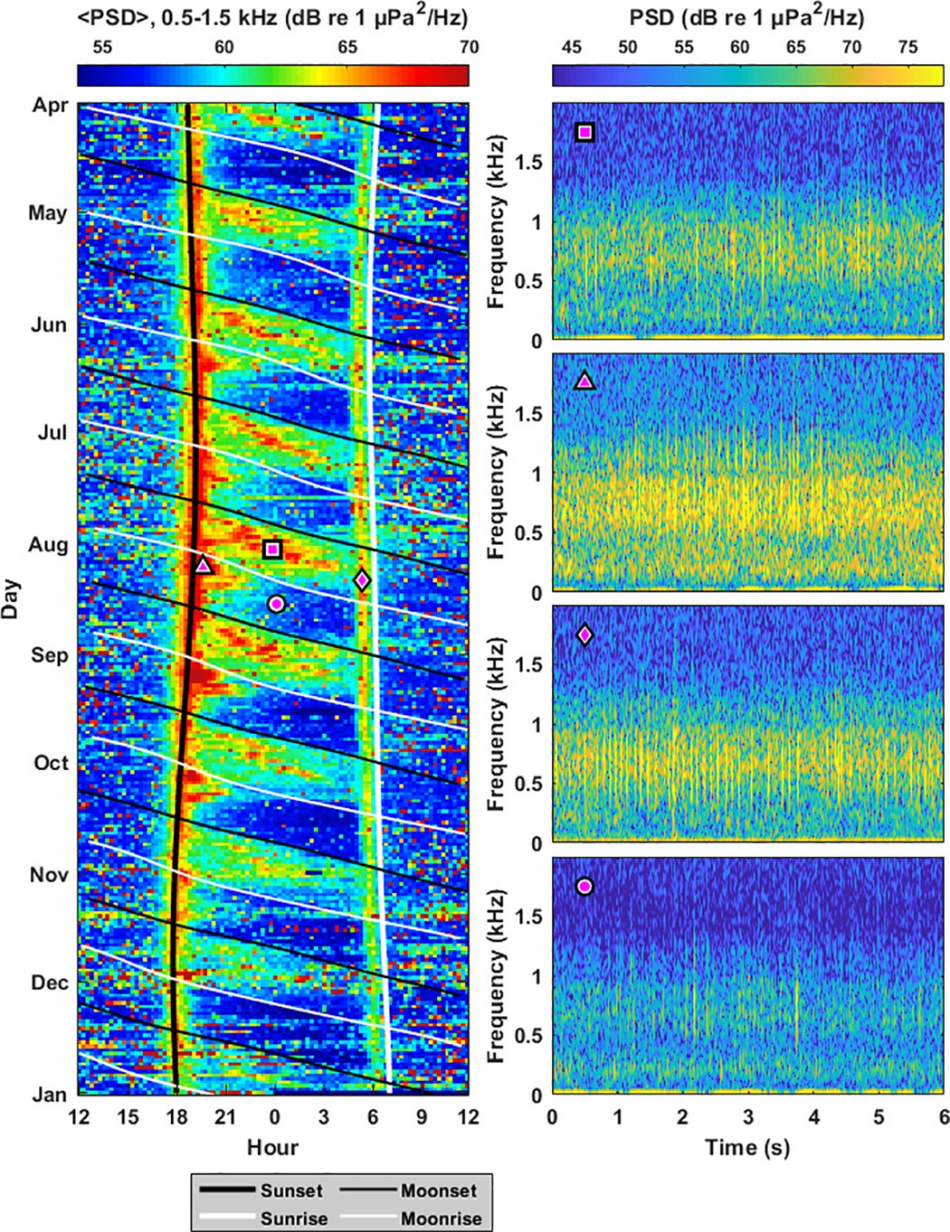
High frequency pulse trains from fish. Significant increases in biological sound at Survey Site 1 are observed during moonlight hours at frequencies where high frequency pulse trains from fish are prominent (0.5–1.5 kHz). The average power spectral density in this frequency band 〈PSD〉 is shown as a function of hour of day and time of year between April and December 2020 (left). Signficant increases in 〈PSD〉 are observed during nighttime hours between moonrise (diagonal thin white lines) and moonset (diagonal thin black lines). There are also significant increases in 〈PSD〉 at sunset (thick vertical black line) and approximately one hour before sunrise (thick vertical white line). Overlain magenta shapes correspond to spectrograms of representative pulse trains (right), where the magenta circle corresponds to a time with reduced biological activity.

**Fig 3:**
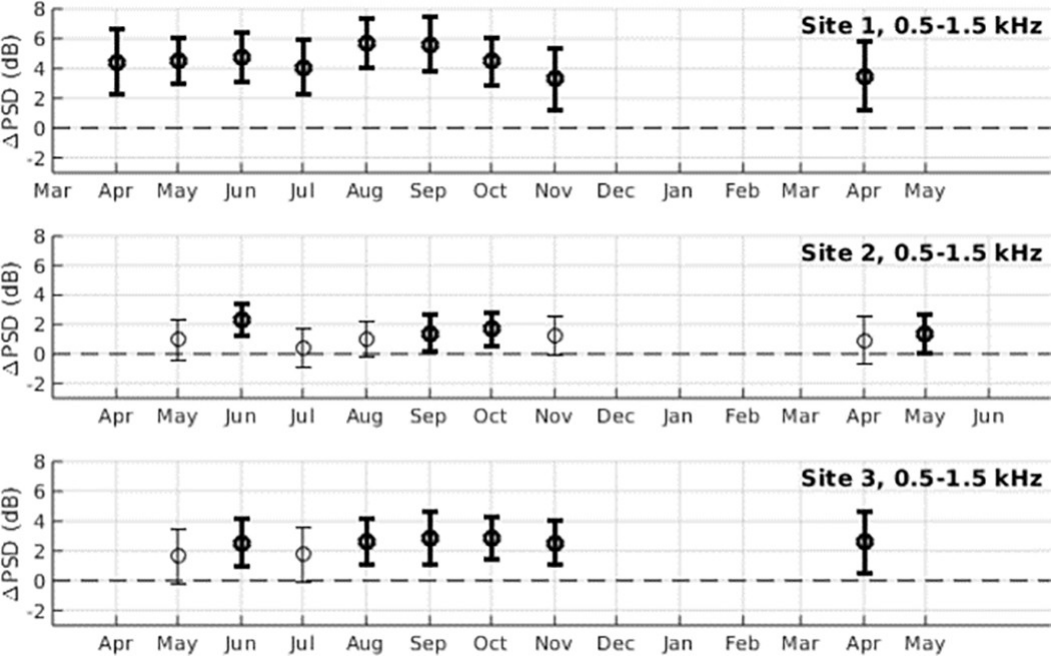
Changes in power spectral density due to high frequency pulse trains. Changes in power spectral density due to the response of marine life to moonlight (ΔPSD) is shown for the frequency band associated with high frequency pulse trains from fish (0.5–1.5 kHz) for months between April 2020 and May 2021. Error bars denote standard deviation, and bolded values represent measurements where the mean is greater than the standard deviation (〈ΔPSD〉 > *σ*_ΔPSD_). Measurements are not made between December and March since humpback whale vocalizations dominated the 0.5–1.5 kHz band in these months.

Coral reef fish such as pomacentrids, mullids, and chaetodontids produce high frequency pulses and pulse train sounds in the 0.5–1.5 kHz band [16]. In all three survey sites studied, there are significant increases in power spectral density during moonlight hours in this band. The response of high frequency pulse trains to moonlight was strongest in Survey Site 1 (Fig 2), where a statistically significant increase in power spectral density is observed during moonlight hours for every month surveyed (Fig 3, S1 Table), with 〈ΔPSD〉 values ranging from 3.28 to 5.71 dB. Statistically significant 〈ΔPSD〉 values were also observed in 4 out of the 9 surveyed months in Site 2, and 6 out of the 8 surveyed months in Site 3.

**Fig 4:**
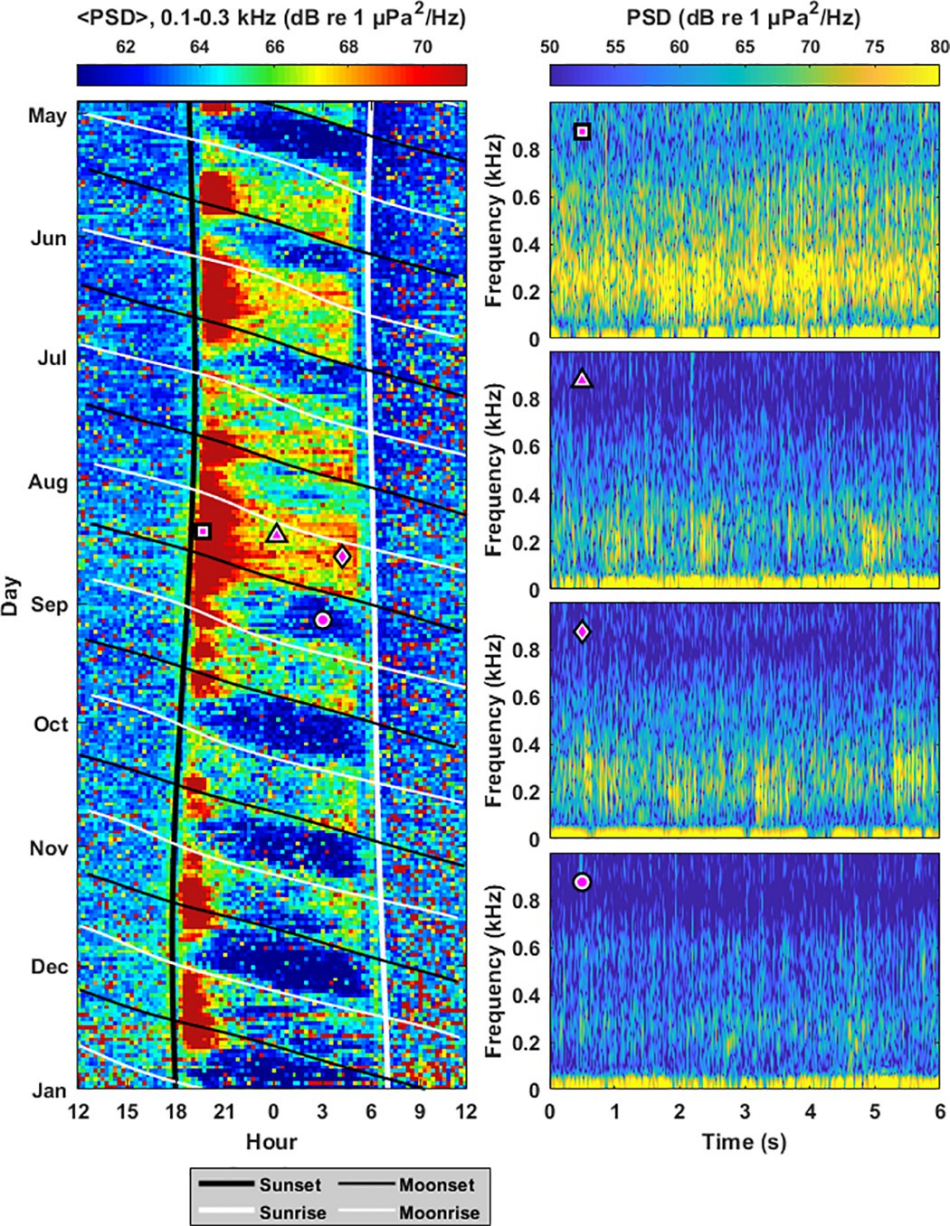
Fish vocalizations. Significant increases in biological sound at Survey Site 2 are observed during non-moonlight hours at frequencies where fish vocalizations are prominent (0.1–0.3 kHz). The average power spectral density in this frequency band 〈PSD〉 is shown as a function of hour of day and time of year between May and December 2020 (left). There are significant increases in 〈PSD〉 during nighttime hours between moonset (diagonal thin black lines) and moonrise (diagonal thin white lines). There are also periodic but significant increases in 〈PSD〉 in the 2–4 hours after sunset (thick vertical black line). Overlain magenta shapes correspond to spectrograms of representative fish vocalizations (right), where the magenta circle corresponds to a time with fewer fish vocalizations.

**Fig 5:**
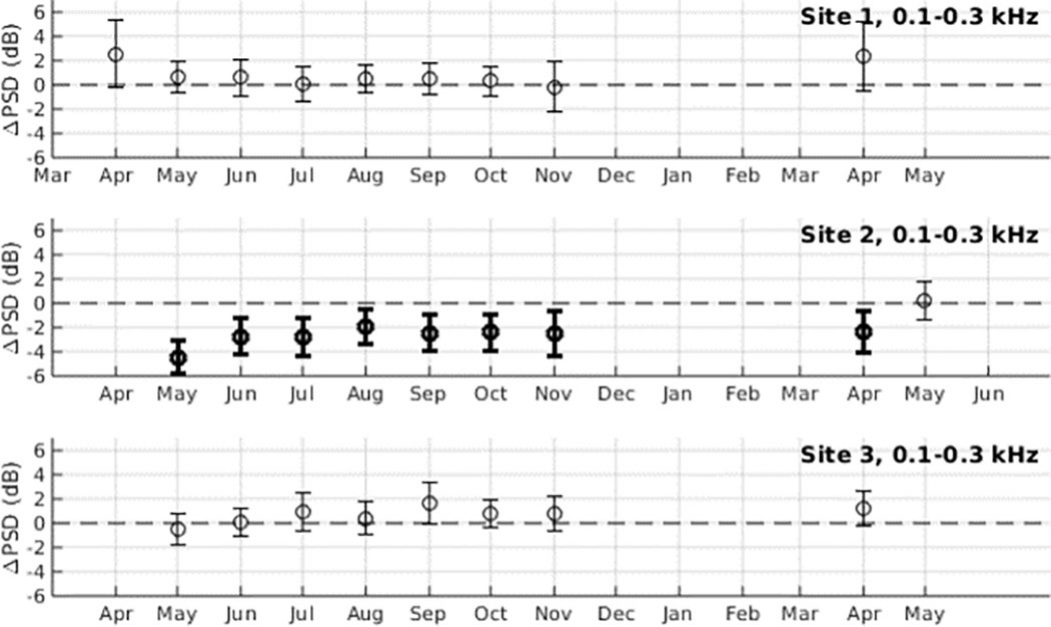
Changes in power spectral density due to fish vocalizations. Changes in power spectral density due to the response of marine life to moonlight (ΔPSD) is shown for the frequency band associated with fish vocalizations (0.1–0.3 kHz) for months between April 2020 and May 2021. Error bars denote standard deviation, and bolded values represent measurements where the mean is greater than the standard deviation (〈ΔPSD〉 > *σ*_ΔPSD_). Measurements are not made between December and March since humpback whale vocalizations dominated the 0.1–0.3 kHz band in these months.

Vocalizations from fish such as holocentrids, pomacentrids, and acanthurids are prominent in Hawiian coral reefs at frequencies between 0.1–0.3 kHz [16]. In Survey Site 2, there are statistically significant decreases in power spectral density in this frequency band during moonlight hours in 8 out of the 9 months surveyed, with 〈ΔPSD〉 values ranging from -1.93 to -4.46 dB (Figs 4 and 5). There were no statistically significant changes in this frequency band during moonlight hours in Sites 1 and 3 for any of the months surveyed (Fig 5, S2 Table).

**Fig 6:**
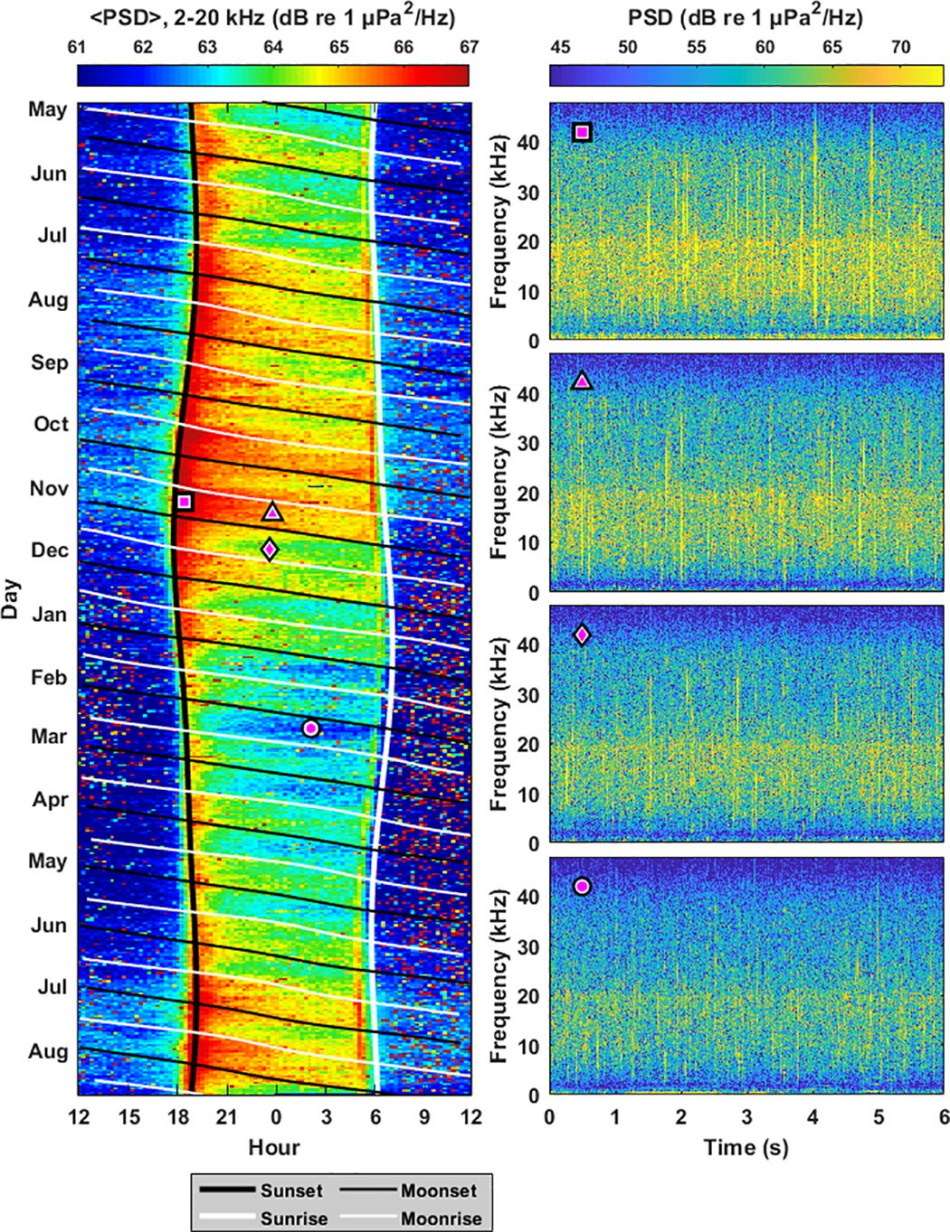
Invertebrate sounds. Significant increases in biological sound at Survey Site 3 are observed during non-moonlight hours at frequencies where invertebrate sounds are prominent (2–20 kHz). The average power spectral density in this frequency band 〈PSD〉 is shown as a function of hour of day and time of year between May 2020 and August 2021 (left). 〈PSD〉 is significantly higher during nighttime hours with a peak at sunset (thick vertical black line) and a less-prominent peak at sunrise (thick vertical white line). There are significant increases in 〈PSD〉 during nighttime hours between moonset (diagonal thin black lines) and moonrise (diagonal thin white lines). Overlain magenta shapes correspond to spectrograms of representative invertebrate clicks (right), where the magenta circle corresponds to a time with reduced invertebrate noise.

**Fig 7:**
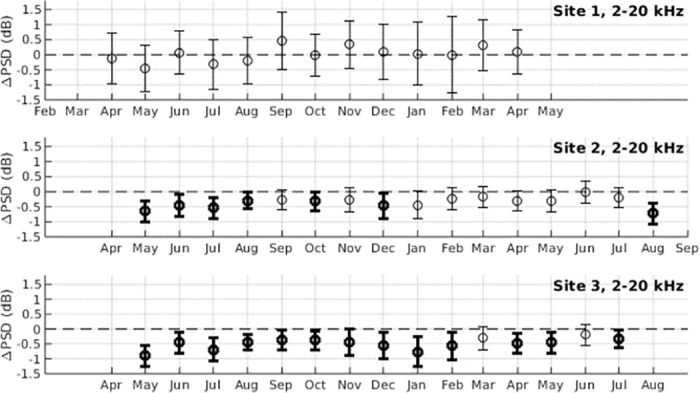
Changes in power spectral density due to invertebrate sounds. Changes in power spectral density due to the response of marine life to moonlight (ΔPSD) is shown for the frequency band associated with invertebrate sounds (2–20 kHz) for months between April 2020 and August 2021. Error bars denote standard deviation, and bolded values represent measurements where the mean is greater than the standard deviation (〈ΔPSD〉 > *σ*_ΔPSD_).

Broadband transient sounds from invertebrates such as snapping shrimp, lobsters, crabs, sea urchins, and bivalves typically occupy frequencies between 2–20 kHz [7, 19–24]. The response of invertebrate sounds to moonlight was strongest in Survey Site 3 (Fig 6), where a statistically significant decrease in power spectral density is observed during moonlight hours for 13 out of the 15 months surveyed, with 〈ΔPSD〉 values ranging from -0.33 to -0.89 dB. Statistically significant 〈ΔPSD〉 values were observed for 7 out of 16 months surveyed in Site 2, and none of the months surveyed in Site 1 (Fig 7, S3 Table).

**Fig 8:**
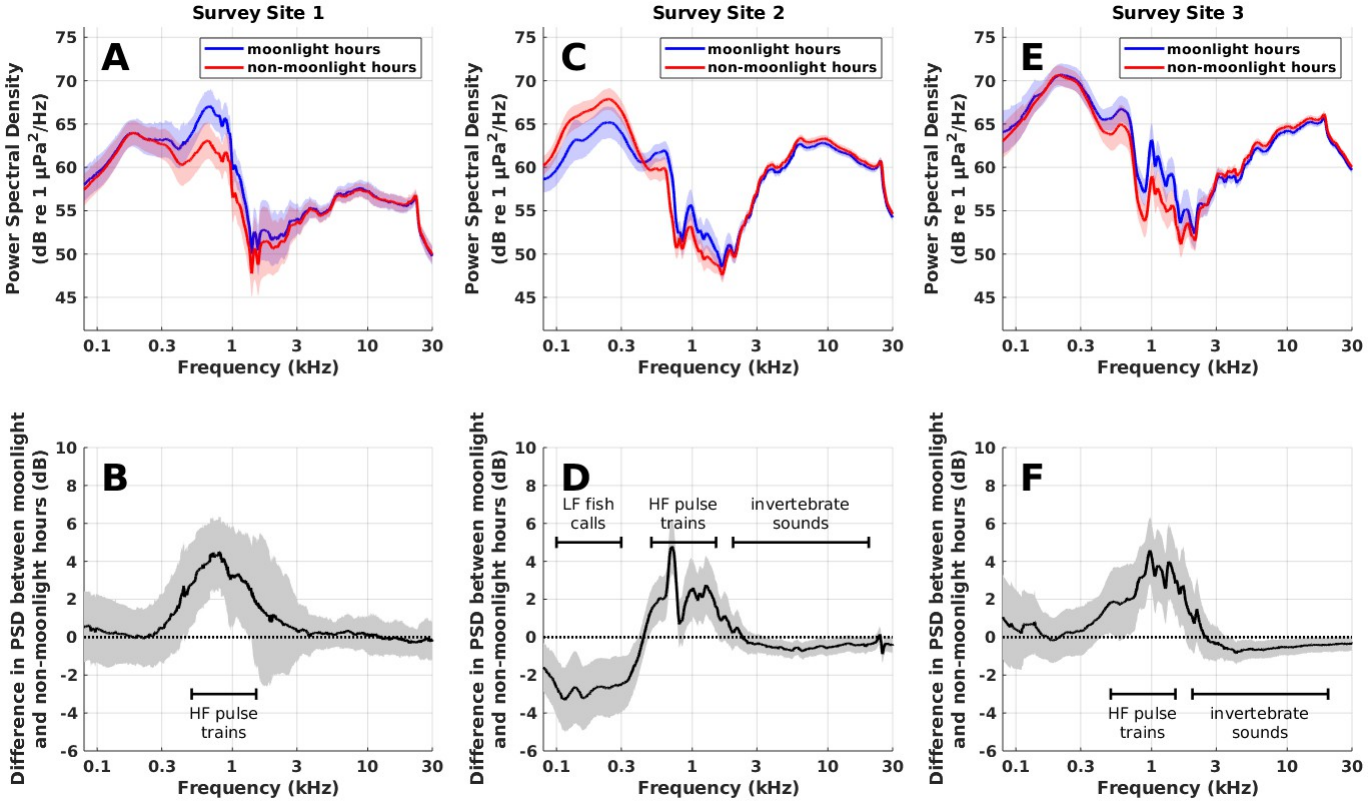
Changes in ambient noise spectrum during moonlight hours. Changes in the spectrum of ambient noise during moonlight hours are shown in June 2020, a month where significant changes in biological noise are observed in all three frequency bands studied. Blue and red lines in A, C, and E designate the average power spectral density (PSD) of ambient noise during nighttime moonlight hours and nighttime non-moonlight hours, respectively. The change in power spectral density due to the response of marine life to moonlight (black lines in B, D, and F) is then calculated as the difference between power spectral density during nighttime moonlight hours (blue lines) and nighttime non-moonlight hours (red lines). Increases in PSD at 0.5–1.5 kHz during moonlight hours (B,D,F) are caused by high frequency pulse trains from fish. Decreases in PSD at 2–20 kHz during moonlight hours (D,F) are caused by reductions in invertebrate sounds. The decrease in PSD during moonlight hours at 0.1–0.3 kHz (D) is caused by reductions in fish vocalizations. Shaded regions in each subplot designate standard deviation.

The combined response of high frequency pulse trains, low frequency fish vocalizations, and invertebrate sounds to moonlight led to significant changes in the spectrum of ambient noise in the Hawaiian coral reefs studied. The spectrum of ambient noise during moonlight hours and non-moonlight hours is shown in Fig 8 for June 2020, a month where significant changes in biological noise are observed in all three frequency bands studied. In Survey Site 1, there are increases in the 0.5–1.5 kHz band during moonlight hours associated with high frequency pulse trains (Fig 8A and 8B). In Site 2, there are increases in the band associated with high frequency pulse trains and decreases in the bands associated with fish vocalizations (0.1–0.3 kHz) and invertebrates (2–20 kHz) (Fig 8C and 8D). In Site 3, there are increases in the band associated with high frequency pulse trains and decreases in the band associated with invertebrates (Fig 8E and 8F).

## Discussion

Biological activity in coral reefs is found to be responsive to changes in nighttime ambient light, with sustained changes in biological sound between moonrise and moonset observed for multiple communities in Hawaiian reefs. High frequency pulse trains were found to be responsive to moonlight in all three survey sites studied, with increases in power spectral density of up to 5.7 dB. Low frequency fish vocalizations were found to be responsive to moonlight in one of the survey sites, with decreases of up to 4.4 dB during moonlight hours. Invertebrate sounds were also found to change with moonlight in two of the sites surveyed, with decreases of up to 0.8 dB. These discoveries suggest that changes in moonlight may trigger nightly shifts in ecosystem interactions in Hawaiian coral reefs.

While previous studies have investigated variations in coral reef sound with respect to monthly cycles in lunar phase [19, 25–28], we show here that reef activity is responsive to the daily rising and setting of the moon. It is possible that these daily lunar cycles give rise to the reported monthly trends in biological sound, since lunar phase is correlated to the percentage of nighttime hours where the moon is above the horizon. For example, the new moon rises close to sunrise and sets close to sunset, so the new moon is almost never above the horizon during nighttime hours. Previous studies showing increases in coral reef fish vocalizations [26–28] and invertebrate sounds [19] during the new moon are therefore consistent with our findings showing increases in these biological sounds during non-moonlight hours. By investigating variations in biological sound over shorter timescales on the order of minutes, we demonstrate that coral reef communities are rapidly responsive to shifts in nighttime ambient light.

Since monthly lunar trends in biological activity have been identified in a variety of coral reef environments, including the Florida Keys [27], the U.S. Virgin Islands [19], the Great Barrier Reef [26], and Maui, Hawaii [28], it is likely that the daily lunar cycles shown here would be similarly present across many reef environments. Understanding these shifts in coral reef soundscapes may be important for ecosystem monitoring and restoration. Changes in the relative abundance of separate reef communities may be detectable by comparing biological soundscapes during moonlight hours and non-moonlight hours. Strategic use of known soundscape variations may also improve efforts to recruit fish and crustacean larva to reefs using artificial playback of healthy soundscapes. Given the significant increase in biological sounds between moonrise and moonset, the response of larva to artificial soundscapes may be affected by whether soundscape recordings were made during moonlight hours.

## Supporting information

S1 Table Mean change in power spectral density due to the response of marine life to moonlight 〈ΔPSD〉 in the band associated with high frequency pulse trains from fish (0.5-1.5 kHz). Values in parentheses are standard deviation (*σ*_ΔPSD_), and measurements where the mean is greater than the standard deviation are bolded. Dashes (–) designate months where hydrophones were not deployed or were only partially deployed. Asterisks (*) designate months where seasonal humpback whale vocalizations dominated the 0.5-1.5 kHz frequency band. Double asterisks (**) designate months where low-frequency electrical noise corrupted the 0.5-1.5 kHz band.
(PDF)

S2 Table Mean change in power spectral density due to the response of marine life to moonlight 〈ΔPSD〉 in the band associated with low frequency fish vocalizations (0.1-0.3 kHz). Values in parentheses are standard deviation (*σ*_ΔPSD_), and measurements where the mean is greater than the standard deviation are bolded. Dashes (–) designate months where hydrophones were not deployed or were only partially deployed. Asterisks (*) designate months where seasonal humpback whale vocalizations dominated the 0.1-0.3 kHz frequency band. Double asterisks (**) designate months where low-frequency electrical noise corrupted the 0.1-0.3 kHz band.
(PDF)

S3 Table Mean change in power spectral density due to the response of marine life to moonlight 〈ΔPSD〉 in the frequency band associated with invertebrate sounds (2-20 kHz). Values in parentheses are standard deviation (*σ*_ΔPSD_), and measurements where the mean is greater than the standard deviation are bolded. Dashes (–) designate months where hydrophones were not deployed or were only partially deployed.
(PDF)

S4 Table The number of one-minute samples available during nighttime moonlight hours (*n*_moon_) and nighttime non-moonlight hours (*n*_no moon_) in each month surveyed. (PDF)

S1 Fig Tidal variations. Measurements of sea level height above the mean in Hawaii are obtained from the NOAA National Buoy Data Center (station 1617433) and plotted against moonrise and moonset. The timing of high and low tide in both locations are not consistently in sync with moonrise (white dots) or moonset (black dots) [29]. Tidal cycles in Hawaii are mixed-semidiurnal, meaning there are two high tides and two low tides of different sizes every lunar day. Black bars correspond to the time between moonset and moonrise. Coordinates of the NOAA buoy in Hawaii are (20.037 N, 155.829 W).
(TIF)

S1 Data (ZIP)

## References

[1] Eddy TD, Lam VWY, Reygondeau G, Cisneros-Montemayor AM, Greer K, Palomares MLD, Bruno JF, Ota Y, Cheung WWL. Global decline in capacity of coral reefs to provide ecosystem services. One Earth. 2021;4,8:1278–1285. doi: 10.1016/j.oneear.2021.08.016

[2] Nedelec SL, Simpson SD, Holderied M, Radford AN, Lecellier G, Radford C, et al. Soundscapes and living communities in coral reefs: temporal and spatial variation. Mar Ecol Prog Ser. 2015;524:125–135. doi: 10.3354/meps11175

[3] Lammers MO, Brainard RE, Au WW, Mooney TA, Wong KB. An ecological acoustic recorder (EAR) for long-term monitoring of biological and anthropogenic sounds on coral reefs and other marine habitats. J Acoust Soc Am. 2008;123:1720–1728. doi: 10.1121/1.2836780 18345859

[4] Freeman LA, Freeman SE. Rapidly obtained ecosystem indicators from coral reef soundscapes. Mar Ecol Prog Ser. 2016;561:69–82. doi: 10.3354/meps11938

[5] Lammers MO, Brainard RE, Au WW, Mooney TA, Wong KB. Coral reef species assemblages are associated with ambient soundscapes. Mar Ecol Prog Ser. 2015;533:93–107. doi: 10.3354/meps11382

[6] Lamont TAC, Williams B, Chapuis L, Prasetya ME, Seraphim MJ, Harding HR, et al. The sound of recovery: Coral reef restoration success is detectable in the soundscape. Journal of Applied Ecology. 2021;59:742–756. doi: 10.1111/1365-2664.14089

[7] Bohnenstiehl DR, Lillis A, Eggleston DB. The Curious Acoustic Behavior of Estuarine Snapping Shrimp: Temporal Patterns of Snapping Shrimp Sound in Sub-Tidal Oyster Reef Habitat. PLOS One. 2016;11. doi: 10.1371/journal.pone.0143691 26761645 PMC4711987

[8] Ferguson SR, Jensen FH, Hyer MD, Noble A, Apprill A, Mooney TA. Ground-truthing daily and lunar patterns of coral reef fish call rates on a US Virgin Island reef. Aquatic Biology. 2022;31:77–87. doi: 10.3354/ab00755

[9] Simpson SD, Meekan MG, McCauley RD, Jeffs A. Attraction of settlement-stage coral reef fishes to reef noise. Mar Ecol Prog Ser. 2004;276:263–268. doi: 10.3354/meps276263

[10] Suca JJ, Lillis A, Kaplan MB, Solow AR, Earl AD, Habtes S, et al. Variable and spatially explicit response of fish larvae to the playback of local, continuous reef soundscapes. Mar Ecol Prog Ser. 2020;653:131–151. doi: 10.3354/meps13480

[11] Tolimieri N, Jeffs A, Montgomery JC. Ambient sound as a cue for navigation by the pelagic larvae of reef fishes. Mar Ecol Prog Ser. 2000;207:219–224. doi: 10.3354/meps207219

[12] Gordon TAC, Radford AN, Davidson IK, Barnes K, McCloskey K, Nedelec SL, et al. Acoustic enrichment can enhance fish community development on degraded coral reef habitat. Nature Communications. 2019;10. doi: 10.1038/s41467-019-13186-2 31784508 PMC6884498

[13] Cato D. Marine biological choruses observed in tropical waters near Australia. J Acoust Soc Am. 1978;64:736–743. doi: 10.1121/1.382038

[14] Staaterman E, Rice AN, Mann DA, Paris CB. Soundscapes from a Tropical Eastern Pacific reef and a Caribbean Sea reef. Coral Reefs. 2013;32:553–557. doi: 10.1007/s00338-012-1007-8

[15] Hawai’i Mapping Research Group. Main Hawaiian Islands Multibeam Bathymetry and Backscatter Synthesis. http://www.soest.hawaii.edu/hmrg/multibeam/bathymetry.php.

[16] Tricas TC, Boyle KS. Acoustic behaviors in Hawaiian coral reef fish communities. Mar Ecol Prog Ser. 2014;511:1–16. doi: 10.3354/meps10930

[17] Au WWL, Banks K. The acoustics of the snapping shrimp *Synalpheus parneomeris* in Kaneohe Bay. J Acoust Soc Am. 1998;103:41–47. doi: 10.1121/1.423234

[18] Kim B, Hahn J, Choi BK, Kim B. Snapping Shrimp Sound Measured Under Laboratory Conditions. Jpn J Appl Phys. 2010;49.

[19] Lillis A, Mooney TA. Snapping shrimp sound production patterns on Caribbean coral reefs: relationships with celestial cycles and environmental variables. Coral Reefs. 2018;37:597–607. doi: 10.1007/s00338-018-1684-z

[20] Coquereau L, Grall J, Clavier J, Jolivet A, Chauvaud L. Acoustic behaviours of large crustaceans in NE Atlantic coastal habitats. Aquatic Biology. 2016;25:151–163. doi: 10.3354/ab00665

[21] Iorio LD, Gervaise C, Jaud V, Robson AA, Chauvaud L. Hydrophone detects cracking sounds: Non-intrusive monitoring of bivalve movement. Journal of Experimental Marine Biology and Ecology. 2012;432–433:9–16. doi: 10.1016/j.jembe.2012.07.010

[22] Radford C, Jeffs A, Tindle C, Mongomery JC. Resonating sea urchin skeletons create coastal choruses. Mar Ecol Prog Ser. 2008;362:37–43. doi: 10.3354/meps07444

[23] Radford CA, Stanley JA, Tindle CT, Montgomery JC, Jeffs AG. Localised coastal habitats have distinct underwater sound signatures. Mar Ecol Prog Ser. 2010;401:21–29. doi: 10.3354/meps08451

[24] Freeman SE, Rohwer FL, D’Spain GL. The origins of ambient biological sound from coral reef ecosystems in the Line Islands archipelago. J Acoust Soc Am. 2014;135:1775–1788. doi: 10.1121/1.4865922 25234977

[25] Tricas TC, Boyle KS. Parrotfish soundscapes: implications for coral reef management. Mar Ecol Prog Ser. 2021;666:149–169. doi: 10.3354/meps13679

[26] McWilliam JN, McCauley RD, Erbe C, Parsons MJG. Patterns of biophonic periodicity on coral reefs in the Great Barrier Reef. Scientific Reports. 2017;7. doi: 10.1038/s41598-017-15838-z 29234024 PMC5727085

[27] Staaterman E, Paris CB, DeFerrari HA, Mann DA, Rice AN, D’Allessandro EK. Celestial patterns in marine soundscapes. Mar Ecol Prog Ser. 2014;508:17–32. doi: 10.3354/meps10911

[28] Kaplan MB, Lammers MO, Zang E, Mooney TA. Acoustic and biological trends on coral reefs off Maui, Hawaii. Coral Reefs. 2018;37:121–133. doi: 10.1007/s00338-017-1638-x

[29] Meteorological and oceanographic data collected from the National Data Buoy Center Coastal-Marine Automated Network (C-MAN) and moored (weather) buoys; 1971. https://www.ncei.noaa.gov/archive/accession/NDBC-CMANWx.

