# Supplementary material for: Moonlight-driven biological choruses in Hawaiian coral reefs
Source: PLoS One. 2024 Mar 20;19(3):e0299916. doi: 10.1371/journal.pone.0299916 (PMC10954159; doi:10.1371/journal.pone.0299916)
Supplement: S1 Table — Values in parentheses are standard deviation (σΔPSD), and measurements where the mean is greater than the standard deviation are bolded. Dashes (–) designate months where hydrophones were not deployed or were only partially deployed. Asterisks (*) designate months where seasonal humpback whale vocalizations dominated the 0.5-1.5 kHz frequency band. Double asterisks (**) designate months where low-frequency electrical noise corrupted the 0.5-1.5 kHz band. (PDF) [file pone.0299916.s001.pdf]

$\langle\Delta\text{PSD}\rangle$ ($\sigma_{\Delta\text{PSD}}$) 0.5-1.5 kHz			
	Site 1	Site 2	Site 3
April 2020	**4.43 (2.16)**	–	–
May 2020	**4.53 (1.56)**	0.96 (1.42)	1.63 (1.81)
June 2020	**4.78 (1.66)**	**2.32 (1.09)**	**2.51 (1.59)**
July 2020	**4.08 (1.84)**	0.43 (1.32)	1.73 (1.86)
August 2020	**5.71 (1.69)**	1.02 (1.24)	**2.64 (1.50)**
September 2020	**5.61 (1.81)**	**1.42 (1.24)**	**2.89 (1.78)**
October 2020	**4.48 (1.59)**	**1.70 (1.15)**	**2.86 (1.38)**
November 2020	**3.28 (2.04)**	1.26 (1.34)	**2.51 (1.48)**
December 2020	*	*	*
January 2021	*	*	*
February 2021	*	*	*
March 2021	*	*	*
April 2021	**3.46 (2.32)**	0.94 (1.65)	**2.58 (2.06)**
May 2021	–	**1.37 (1.28)**	**
June 2021	–	**	**
July 2021	–	**	**
August 2021	–	**	–

Table S1: Mean change in power spectral density due to the response of marine life to moonlight $\langle\Delta\text{PSD}\rangle$ in the band associated with high frequency pulse trains from fish (0.5-1.5 kHz). Values in parentheses are standard deviation ($\sigma_{\Delta\text{PSD}}$), and measurements where the mean is greater than the standard deviation are bolded. Dashes (–) designate months where hydrophones were not deployed or were only partially deployed. Asterisks (*) designate months where seasonal humpback whale vocalizations dominated the 0.5-1.5 kHz frequency band. Double asterisks (**) designate months where low-frequency electrical noise corrupted the 0.5-1.5 kHz band.
