# Supplementary material for: Moonlight-driven biological choruses in Hawaiian coral reefs
Source: PLoS One. 2024 Mar 20;19(3):e0299916. doi: 10.1371/journal.pone.0299916 (PMC10954159; doi:10.1371/journal.pone.0299916)
Supplement: S4 Table — (PDF) [file pone.0299916.s004.pdf]

	Site 1		Site 2		Site 3	
	n_{moon}	$n_{\text{no moon}}$	n_{moon}	$n_{\text{no moon}}$	n_{moon}	$n_{\text{no moon}}$
April 2020	226	259	-	-	-	-
May 2020	209	219	314	333	308	342
June 2020	190	196	285	300	287	302
July 2020	221	198	331	299	324	299
August 2020	265	220	402	323	399	332
September 2020	296	240	450	360	442	355
October 2020	358	259	527	395	538	396
November 2020	351	284	520	436	530	431
December 2020	381	307	560	469	568	460
January 2021	355	317	532	478	533	475
February 2021	250	309	380	463	377	463
March 2021	289	292	435	431	426	437
April 2021	225	262	335	396	339	397
May 2021	-	-	299	351	303	356
June 2021	-	-	267	319	265	328
July 2021	-	-	315	319	321	311
August 2021	-	-	378	342	–	–

Table S4: The number of one-minute samples available during nighttime moonlight hours (n_{moon}) and nighttime non-moonlight hours ($n_{\text{no moon}}$) in each month surveyed.
