# Supplementary figures and images for: Moonlight-driven biological choruses in Hawaiian coral reefs
Source: PLoS One. 2024 Mar 20;19(3):e0299916. doi: 10.1371/journal.pone.0299916 (PMC10954159; doi:10.1371/journal.pone.0299916)

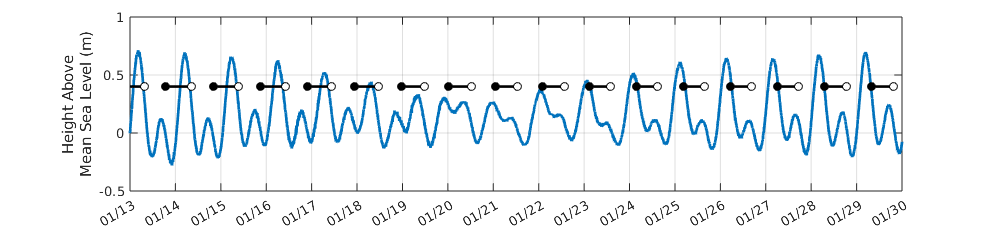

Supplement: S1 Fig — Measurements of sea level height above the mean in Hawaii are obtained from the NOAA National Buoy Data Center (station 1617433) and plotted against moonrise and moonset. The timing of high and low tide in both locations are not consistently in sync with moonrise (white dots) or moonset (black dots) [29]. Tidal cycles in Hawaii are mixed-semidiurnal, meaning there are two high tides and two low tides of different sizes every lunar day. Black bars correspond to the time between moonset and moonrise. Coordinates of the NOAA buoy in Hawaii are (20.037 N, 155.829 W). (TIF) [file pone.0299916.s005.tif]
